# Parenteral administration of twin-bearing ewes with L-arginine enhances the birth weight and brown fat stores in sheep

**DOI:** 10.1186/2193-1801-2-684

**Published:** 2013-12-21

**Authors:** Sue McCoard, Francisco Sales, Nina Wards, Quentin Sciascia, Mark Oliver, John Koolaard, Danitsja van der Linden

**Affiliations:** AgResearch Grasslands, Private Bag 11008, Palmerston North, 4442 New Zealand; Ngapouri Research Farm, Liggins Institute, University of Auckland, Auckland, New Zealand

**Keywords:** Sheep, Fetal, Birth weight, Arginine, Amino acids

## Abstract

The objective of this study was to evaluate the effects of parenteral administration of L-arginine (Arg) to well-fed twin-bearing ewes from day (d) 100 of pregnancy to birth on fetal growth, body composition and neonatal behavior. Ewes received an i.v. bolus of either 345 μmol Arg-HCl/kg bodyweight or saline solution (control) 3 times a day. At d 140 of pregnancy, Arg-supplemented and control ewes were euthanized and fetal weight and fetal organ weight recorded, and maternal and fetal plasma concentrations of amino acids, hormones and metabolites analyzed. A subset of ewes was allowed to lamb and birth weight, body dimensions and behavior of the lambs in the first 2 hours(h) following birth recorded and blood samples collected. At d 140 of pregnancy, fetal weight internal organ weights were unaffected by treatment with the exception of brown fat stores which were increased by 16% in fetuses from Arg-supplemented ewes relative to controls (*P* < 0.05). At birth, there was an interaction (*P* = 0.06) between treatment and sex for birth weight of the lamb. The ewe lambs from Arg-supplemented ewes were 12% (*P* < 0.05) heavier at birth compared with controls whereas birth weight of male lambs did not differ. These results indicate that maternal Arg supplementation enhanced brown fat stores in the fetus and countered some effect of fetal growth restriction due to litter size in female lambs. Increasing birth weight of female lambs and enhancing brown fat stores of all lambs may have important implications for lamb survival and postnatal growth.

## Introduction

Increasing prolificacy in sheep is a cost effective approach to increase the efficiency of meat production in a sustainable manner. Advances in genetic selection and breeding have significantly increased the proportion of multiple-pregnancies. However, competition between twin fetuses in mid-late pregnancy leads to restricted fetal growth, organ and tissue development, lower birth weight and increased mortality compared to their singleton counterparts (McCoard et al. 2000; Wu et al. 2006; Gootwine et al. 2007). Twin-reduction experiments confirm the significance of the effect of constraint in late pregnancy, but also indicate that periconceptional events like twinning lead to a reduced growth trajectory in late pregnancy (Hancock et al. 2012). Mild growth restriction *in utero* as a result of twinning also has carryover effects on postnatal growth and carcass traits at market weight compared to singletons, even when ewes are well-fed (Afolayan et al. 2007; McCoard et al. 2010). Maternal constraint, resulting from decreased uterine capacity to deliver sufficient nutrients, oxygen and space to support multiple fetuses, is postulated to be a major factor limiting the survival and growth of the fetus in humans (Blickstein 2005) and other mammals (Freetly and Leymaster 2004; Gootwine et al. 2007). Furthermore, the increased frequency of human-twin pregnancies has also increased in the last 20 years leading to more premature and low-birth weight infants (Fliegner 1989; Siddiqui and McEwan 2007). Intervention strategies however, are not currently available to counter the effects of growth restriction *in utero* due to multiple-pregnancies.

It is hypothesized that maternal supplementation with arginine (Arg), a common physiological substrate for nitric oxide and polyamine synthesis, may reduce intra-uterine growth restriction either via altering utero-placental blood flow or through direct actions on the developing fetus (Wu et al. 2004). This hypothesis is supported by recent research in sheep which has indicated that maternal parenteral administration of Arg (155 μmol/kg live weight) from d 60 of pregnancy to birth can counter some effects of fetal growth restriction induced by under-feeding (Lassala et al. 2010). Furthermore, Lassala et al. (2011) reported that parenteral administration of Arg (345 μmol/kg live weight) to multiple-bearing ewes between d 100 and 121 of pregnancy increased birth weight of quadruplet lambs by 23% relative to un-treated controls, while the birth weight of triplet- and twin-born lambs was similar for parental Arg supplemented and unsupplemented animals (Lassala et al. 2011). We have previously reported that fetal growth restriction in twin sheep is not observed until approximately d 115–120 of pregnancy compared to singletons (McCoard et al. 2000). This could explain the lack of effect of maternal Arg supplementation on fetal growth in twins observed in the study by Lassala et al. (2011) who terminated the Arg supplementation at d 121 of pregnancy. We hypothesized that maternal parenteral administration of Arg to well-fed twin-bearing ewes from d 100 of pregnancy to birth would increase offspring birth weight.

## Materials and methods

### Ewes

Multiparous Romney ewes were synchronized and naturally mated to one of two Poll Dorset sires to minimize the paternal genetic effects on size and weight of the fetuses. Ewes were mated in 3 separate groups. Ewes in cohort 1 (slaughtered at d 140 of pregnancy (P140); *n* = 23) were mated in 2 groups, 3 weeks apart, whereas cohort 2 (*n* = 25), which were allowed to lamb, were mated as a single group approximately 1 month later. The live weight of the ewes at mating was 65–75 kg with a body condition score of 3–3.5 (1–5 scale; (Jefferies 1961)). Twin-bearing ewes were identified at d 60 post-mating via transabdominal ultrasonagraphy. From mating to pregnancy diagnosis, ewes were managed under commercial grazing conditions to meet or exceed nutritional requirements. All ewes were exposed to a lucerne-based pellet diet (University B mix, Camtech Nutrition, Cambridge NZ) by providing up to 20% of daily requirements while grazed on pasture for 2 weeks prior to indoor housing. At P80, ewes were acclimatized to indoor housing in group pens and lucerne-based pellet diet only, formulated to meet 100% of NRC-recommended maintenance requirements for twin-bearing pregnant ewes (National Research Council 1985), with water freely available. Ewes were fed once daily (between 0800 and 0900 h). On P90, ewes were moved to individual pens with mesh sides to facilitate visualization with other animals. The diet contained 6.69 mg/g of Arg (6% of total amino acids). Gross composition of the diet (Table 1) was determined using near-infrared spectroscopy (FeedTech, AgResearch Grasslands, Palmerston North, NZ). Macro-element composition of the diet (Table 1) was determined by Plasma Emission Spectrometry (Eurofins NZ Labs, Ruakura, NZ). Ewes were weighed and body condition scored weekly (Jefferies 1961), and feed allowances were adjusted according to live weight changes (National Research Council 1985). Daily feed intake was recorded by weight of refusals. The animal manipulations were performed at Ngapouri Research Farm Laboratory, Reporoa, NZ and approved by the University of Auckland Animal Ethics Committee (C889).

**Table 1:** **Composition of the lucerne-based diet fed to the ewes during the duration of the trial**

	Content
Metabolizable energy (MJ/kg dry matter)	10.04
Dry matter (DM; %)	86.9
Composition (%DM)	
Crude protein	17.1
Lipid	2.9
Ash	3.9
Acid detergent fibre	20.9
Neutral detergent fibre	41.9
Soluble sugars and starch	11.7
Macro element Composition (%DM)	
Potassium	2.01
Magnesium	0.29
Phosphorus	0.38
Calcium	1.25
Sulphur	0.33
Sodium	0.40

### Experimental design

Ewes were randomly assigned to Arg and control (saline supplementation) groups at P95. At P97, polyvinyl catheters were inserted into the tarsal vein of the hind leg under brief anesthesia induced by injectable anaesthetic and sedation (combination of 0.5 mg/kg body weight (BW) Pamlin and 10 mg/kg body weight Ketamine, Parnell Technologies, Auckland, NZ). The externalized catheter was flushed with heparinized saline (0.9% sodium chloride, Baxters Healthcare Pty Ltd, Old Toongabbie, Australia; 10 U/mL sodium heparin), sealed with stopcocks, secured in a plastic bag and anchored to the mid-line of the ewe. The bag and catheter were further secured with tube net. After catheter insertion, the ewes received prophylactic i.m. antibiotics (Duplocillin® LA, Intervet LTD, Newmarket, Auckland NZ, 2 mL/50 kg BW).

From P100 to P140 (cohort 1; *n* = 23) or P100 to birth (cohort 2; *n* = 25) ewes received either a bolus of Arg-mono-hydrochloride (L-Arg-HCL; Merck KGaA, Darmstadt, Germany; 345 μmol/kg BW) or approximately the same volume of sterile saline, three times daily (0800, 1600, 2400 h). The chosen Arg dose rate was based on the previous study by Lassala et al. (2011).

The Arg solution was prepared fresh each day using sterile physiological saline (0.9% sodium chloride, Baxters Healthcare Pty Ltd, Australia) with a final concentration of 1.8 g Arg per 5 mL and pH adjusted to 7.0 with 1 mol/L NaOH. Each individual bolus was passed through a 0.22 μm PES syringe filter (Jet Biofilt, Elgin IL, USA) directly into the catheter to ensure the solution was free of microbial pathogens. Between boluses, the catheters were flushed with heparinized saline (10 U/mL sodium heparin) to maintain patency and prevent clotting. For cohort 2, administration of treatment was ceased at the first signs of labor which was between 1 and 10 h prior to birth.

At P120, ewes of both cohorts were blood sampled prior to (-5 minutes (min)) and 30, 60 and 180 min after Arg administration, using jugular venipuncture to determine the change in plasma amino acid concentrations over time. Plasma was separated and stored at -20°C until analyzed for amino acids, metabolites and hormones.

At P140, ewes of cohort 1 were blood sampled one h following Arg administration, just prior to euthanasia. After euthanasia of the ewes with 0.5 mL/kg BW Pentobarb 300 (Provet NZ Pty Ltd, Auckland, NZ) the abdominal cavity was opened to remove the gravid uterus, and opened to expose the fetuses. A blood sample from each fetus (cardiac puncture) was collected, followed by clamping of the umbilical cord and removal of the fetuses from the uterus. Weight of each fetus, sex, crown-rump-length and thoracic-girth circumference, lengths of front and hind legs and weights of the internal organs were recorded. Fetal brown fat weight was defined as the weight of all peri-renal adipose tissue (which at this stage in development is brown adipose tissue (Alexander 1978)). From the 23 ewes available for cohort 1, three ewes were omitted from the study. One ewe was omitted due to a defective catheter (treatment) and two ewes (one treatment and one control) aborted their lambs prior to day 140 of pregnancy for unknown reasons. Thus, in total 20 ewes were used in cohort 1 (Arg *n* = 9; control *n* = 11).

The ewes of cohort 2 (Arg *n* = 13; control *n* = 12) were allowed to lamb naturally. One ewe (control) from the original cohort of 26 was omitted from the study due to aborting her lambs for unknown reasons. Ewes were continuously monitored during the lambing period to identify any potential lambing difficulties and to observe and record lamb behaviours within the first 2 h after birth. The following lamb behaviours were recorded: time (recorded in min) taken to kneel, shake their head, try to stand, successfully stand, reach the udder, first unsuccessful suckle, first successful suckle, and the percentage of lambs that fed unassisted within 2 h of birth as previously reported by (Dwyer et al. 2005). Assistance with lambing was provided when required. Within 2 h of birth, and following their first colostrum feed, ewe and lambs were blood sampled and lambs were weighed, ear-tagged and body dimensions (crown-rump length, thoracic-girth circumference and front and hind leg length) recorded.

### Determination of amino acids, other metabolites and hormones in plasma

Plasma samples from randomly selected Arg and control ewes from cohort 1 and 2 at P120 (*n* = 9/group), P140 (*n* = 6/group) and 2 h post-birth (*n* = 5/group) were selected for analysis. Similarly, plasma samples from randomly selected P140 fetuses (*n* = 6/group; 3 female and 3 males) and lambs at 2 h post-birth (*n* = 10/group; 5 females and 5 males) were selected for analysis. Amino acids were determined by ion-exchange chromatography as previously described (van der Linden et al. 2013). All plasma samples were analyzed for glucose, free fatty acids (NEFA), triglyceride, insulin and insulin-like growth factor-I (IGF-I) concentrations. Plasma metabolite concentrations were measured using a Hitachi 902 autoanalyzer (Hitachi High Technologies Corporation, Tokyo, Japan) using commercial kits for glucose and triglyceride (intra-assay CV 1.2% and 10.8% respectively) (Roche, Mannheim, Germany) and NEFA and glycerol (intra-assay CV 3.7% and 5.3%, respectively) (Randox Laboratories Ltd, Ardmore, Crumlin, UK). Plasma IGF-I concentrations were measured by specific radioimmunoassay (RIA) using an IGFBP-blocked RIA (Blum and Breier 1994; Vickers et al. 1999) with an inter-assay variation of 5.75% and intra-assay variation of 2.87%. The method was established and validated for maternal and fetal sheep plasma. Insulin was measured by RIA, with ovine insulin as the standard (Sigma, Batch no. I9254). The minimum detectable concentration was 0.02 ng/mL and the inter-assay CV was 11.6%.

### Statistical analyses

Results are expressed as means ± standard error of the mean (SEM). The data were analysed using the NLME package in R (R Core Team 2012). Linear mixed effects models were fitted and included the fixed effect of treatment group (Arg vs. control) for maternal data. The model for lamb/fetal data included the fixed effects of treatment group, sex of lamb (female vs. male), treatment by sex interaction, and the random effects of ewe to adjust for the twinning effect, and in the case of cohort 1, for mating subgroup to account for any differences due to time of mating. Fetal weight was fitted as a covariate in the analysis of body dimensions and organ weight at P140. Treatment by sex interaction for body dimension and organ weight parameters were not significant, therefore only the main effects are presented. The percentage of lambs that needed assistance with feeding 2 h after birth were analyzed using the GENMOD procedure (SAS Institute Inc. 2011) for binomial data with the fixed effect of treatment group.

## Results

### Feed intake and body weight and condition of ewes

**Figure 1:**
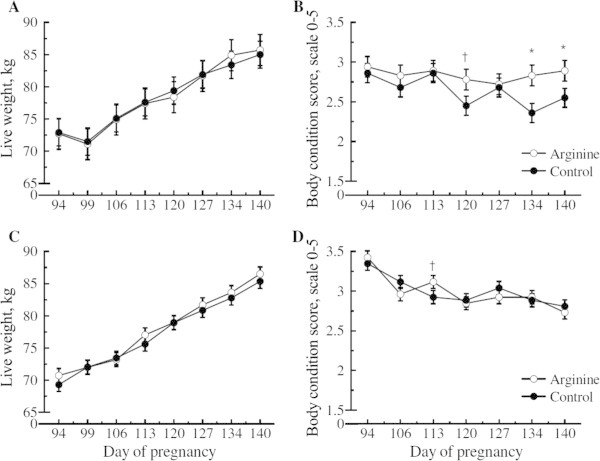
Live weight and body condition score from day 94 to 140 of pregnancy of ewes either supplemented with arginine or saline (Control) from day 100 to 140 of pregnancy are presented as least square means ± SE where * and † indicate the treatment effect at ***P*** **< 0.05 and** ***P*** **< 0.10 respectively. (A)** Cohort 1, P140 live weight **(B)** Cohort 1 P140 body condition score **(C)** Cohort 2 live weight at birth **(D)** Cohort 2 body condition score at birth.

Average daily food intake of the ewes was not affected by Arg relative to controls in either cohort 1 (2.3 ± 0.1 kg vs. 2.3 ± 0.1 kg; *P* = 0.93 for Arg and control ewes), or cohort (2.5 ± 0.2 kg vs. 2.6 ± 0.2 kg; *P* = 0.23 for Arg and control ewes). Arginine did not affect ewe live weight in cohort 1 or cohort 2 throughout pregnancy (Figure 1A and C). However, body condition score of ewes in cohort 1 was affected, such that Arg ewes tended (*P* = 0.06) to have greater body condition scores on P120 and this difference was significant at P134 (*P* = 0.007) and P140 (*P* = 0.05) relative to control ewes (Figure 1B). In cohort 2, Arg ewes tended (*P* = 0.10) to have greater body condition scores on P113 relative to control ewes (Figure 1D).

### Fetal and lamb body weight, dimensions and fetal organ weight

Maternal Arg supplementation had no effect on fetal weight or body dimensions at P140 apart from front-leg length which tended to be longer for fetuses of Arg ewes compared to controls (Table 2). Arginine supplementation resulted in a 16% increase in fetal brown fat stores relative to control fetuses, whereas no other differences in fetal organ weight were observed between the treatment groups (Table 2). The length of the small intestine did not differ between control and Arg fetuses (data not shown). At P140, male fetuses were heavier than female fetuses in both treatment groups, and this effect remained after adjusting for maternal weight (5.6 ± 0.1 vs. 5.0 ± 0.1 kg; *P* < 0.0001).

**Table 2:** **Body weight, skeletal dimensions, organ weights and digestive tract weight of fetuses at d 140 of pregnancy (P140) from twin-bearing ewes either supplemented with arginine or saline (control) from P100 to P140** ^**1**^

		Arginine	Control	SEM	***P***value
n		18	22		
Body weight (kg) and dimensions^2^ (cm)					
	Fetal weight	5.2	5.4	0.2	0.53
	Crown rump length	59.6	59.6	0.5	0.83
	Thoracic girth	36.4	35.9	0.2	0.13
	Front leg length	32.3	31.8	0.2	0.07
	Hind leg length	38.0	37.5	0.3	0.17
Organ weight (g)^2^					
	Heart	38.6	36.4	1.5	0.28
	Lungs	163.1	154.5	5.1	0.25
	Liver	132.5	134.5	4.3	0.75
	Spleen	7.8	7.9	0.5	0.82
	Right Kidney	12.6	12.7	0.4	0.95
	Left Kidney	12.0	12.3	0.3	0.48
	Perirenal fat	8.9	7.7	0.4	0.03
	Thymus	32.0	29.9	1.4	0.30
	Lymph node	1.27	1.25	0.2	0.95
	Thyroid glands	1.8	1.8	0.1	0.80
	Adrenal glands	0.7	0.7	0.1	0.56
Digestive tract weight (g) ^2^					
	Stomach	29.4	27.3	2.5	0.55
	Large intestine	18.7	18.4	0.7	0.78
	Small intestine	104.3	106.2	3.8	0.72

^1^Data are presented as means (predicted from the fitted statistical model) and average SEM, together with *P* value for treatment effect. ^2^With the exception of fetal weight, data were analyzed with fetal weight as covariate.

**Figure 2:**
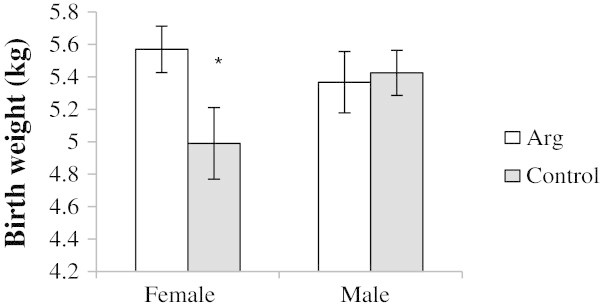
**Birth weight of lambs born to ewes supplemented with arginine (Arg;** ***n*** **= 17 females and** ***n*** **= 9 males) or saline (control;** ***n*** **= 5 females and** ***n*** = 17 males) pre presented as means ± SEM where * indicates the treatment effect ( ***P*** **< 0.05).**

At birth, there was a tendency for an interaction (*P* = 0.06) between Arg and lamb sex for birth weight, such that ewe lambs from Arg ewes were 12% heavier (*P* < 0.05) at birth compared to control female lambs (Figure 2). Control male lambs tended to be 9% heavier than control female lambs at birth (5.4 ± 0.2 vs. 5.0 ± 0.2 kg, *P* = 0.08). However, there was no difference in birth weight between male and female lambs born to Arg ewes (5.4 ± 0.2 vs. 5.6 ± 0.1 kg, *P* = 0.40). Rectal temperatures of lambs born to Arg ewes were higher than that of lambs born to control ewes (39.9 ± 0.16 vs. 39.3 ± 0.16°C; *P* = 0.02), two h post-birth. Maternal Arg supplementation had little effect on crown-rump length of the offspring (data not shown). However, a treatment by sex interaction was observed for thoracic-girth circumference, such that female lambs born to Arg ewes tended to have a greater thoracic girth compared to female lambs born to control ewes (41.8 ± 0.5 vs. 40.1 ± 0.8 cm; *P* = 0.07). No effects of treatment on crown-rump-length or thoracic girth circumference were observed between male offspring, nor were there any effects of treatment on front or hind leg length (data not shown).

### Concentrations of amino acids in maternal plasma

**Figure 3:**
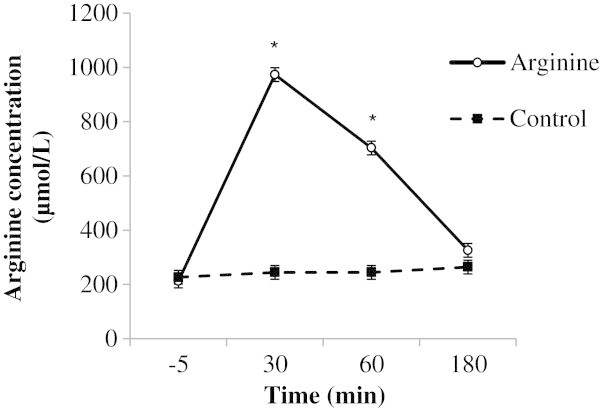
Plasma arginine concentrations immediately prior to (-5 min) and 30, 60 and 180 minutes following an iv. bolus of arginine or saline (controls) in twin-bearing ewes (n = 9/group; Group 1 and 2 ewes represented) at 120 days gestation presented as means ± standard error of the mean (SEM) where * indicates the treatment effect (P < 0.05).

At P120, Arg concentrations were elevated by 5-fold relative to controls 30 min after the Arg administration and remained elevated 3-fold until at least 60 min post-infusion (Figure 3). No difference between treatment groups was observed immediately prior to the Arg administration. A treatment by time interaction was observed for citrulline, methionine and ornithine concentration (Table 3). A divergence in the concentrations of citrulline and methionine concentration was evident within 30 min of Arg administration, such that control ewes had higher concentrations of citrulline and methionine than Arg ewes. In contrast, concentrations of ornithine were increased in Arg ewes within 30 min of Arg administration and reached a peak by 60 min followed by a decline to 180 min, but still remained elevated relative to saline-treated controls. Glycine and histidine concentrations were decreased relative to controls at all time points after Arg administration.

**Table 3:** **Plasma concentrations of amino acids (μmol/L) -5, 30, 60 and 180 min (T) after supplementation (Trt) at d 120 of pregnancy (P120) of twin-bearing ewes either supplemented with arginine (** ***n*** **= 9) or saline (control;** ***n*** **= 9) from P100 to P140 or parturition** ^**1**^

	-5 min		30 min		60 min		180 min		SEM	***P***value		
	Arginine	Control	Arginine	Control	Arginine	Control	Arginine	Control		Trt	T	Trt × T
Taurine	67	81	83	93	91	101	83	120	11	0.12	<0.01	0.24
Aspartic Acid	7	8	8	10	9	9	9	8	1	0.47	0.19	0.48
Threonine	154	176	156	222	173	193	183	221	20	0.09	0.15	0.47
Serine	95	127	114	125	106	121	116	136	13	0.11	0.60	0.84
Asparagine	27	56	43	45	35	43	35	46	10	0.13	0.95	0.53
Glutamic acid	74	85	85	74	101	84	72	64	9	0.41	0.06	0.43
Glutamine	266	289	273	288	317	316	314	296	24	0.79	0.28	0.82
Glycine	454	532	454	559	471	572	475	587	25	<0.01	0.04	0.64
Alanine	217	234	215	246	226	243	199	231	15	0.16	0.13	0.70
Citrulline	126	150	123	155	135	166	144	192	13	0.04	<0.01	0.03
Valine	247	256	256	269	258	301	263	290	20	0.32	0.21	0.66
Cystine	2	1	2	3	13	5	12	2	5	0.39	0.26	0.56
Methionine	30	36	29	38	29	39	25	40	2	<0.01	0.75	0.04
Isoleucine	133	126	137	137	144	157	127	139	11	0.71	0.10	0.64
Leucine	193	183	200	203	210	214	172	211	15	0.59	0.13	0.14
Tyrosine	75	68	77	76	79	78	74	80	6	0.85	0.25	0.33
Phenylalanine	64	75	64	70	66	69	60	68	5	0.16	0.49	0.76
Tryptophan	62	62	46	43	67	56	56	62	5	0.54	<0.01	0.40
Ornithine	145	150	313	151	346	151	249	158	14	<0.01	<0.01	<0.01
Lysine	154	183	208	205	216	229	184	233	17	0.24	<0.01	0.25
Histidine	124	161	133	179	117	141	97	147	13	<0.01	0.02	0.70
Proline	95	108	96	113	103	112	112	114	7	0.15	0.16	0.54

^1^Data are presented as means and average SEM, together with *P* values for treatment and time effects and their interaction.

At P140, Arg ewes had elevated concentrations of Arg and ornithine, whereas concentrations of serine, glycine and methionine were reduced compared to controls (Table 4). Within 2 h post-birth, Arg ewes had decreased concentrations of methionine and histidine, and increased concentrations of alanine and ornithine compared to saline-treated control ewes.

**Table 4:** **Plasma concentrations of amino acids (μmol/L) 1 h after supplementation at d 140 of pregnancy (P140) or 2 h post-birth of twin-bearing ewes either supplemented with arginine or saline (control) from P100 to P140 or parturition** ^**1**^

	Ewe plasma							
	P140				2 h post-birth			
	Arginine	Control	SEM	***P***value	Arginine	Control	SEM	***P***value
*n*	6	6			5	5		
Taurine	61	77	12	0.34	63	74	15	0.63
Aspartic acid	6	7	1	056	6	5	1	0.71
Threonine	148	235	30	0.07	91	130	17	0.15
Serine	66	91	8	0.05	52	53	7	0.88
Asparagine	24	44	8	0.13	13	17	2	0.36
Glutamic acid	80	65	13	0.40	53	63	10	0.50
Glutamine	257	266	13	0.60	191	218	13	0.19
Glycine	516	795	58	<0.01	379	396	36	0.74
Alanine	169	167	14	0.86	200	247	27	0.24
Citrulline	211	262	32	0.29	194	156	23	0.27
Valine	227	223	27	0.91	143	168	33	0.61
Methionine	26	38	2	<0.01	20	29	2	0.01
Isoleucine	113	114	11	0.88	105	78	42	0.67
Leucine	161	164	18	0.92	84	124	25	0.29
Tyrosine	57	64	4	0.27	49	39	10	0.50
Phenylalanine	55	62	5	0.30	40	54	11	0.38
B-Alanine	1	0.5	0.5	0.64	200	247	27	0.24
Tryptophan	59	63	5	0.63	55	48	12	0.66
Ornithine	394	137	38	<0.01	113	62	12	0.01
Lysine	212	192	17	0.44	121	119	14	0.94
Histidine	91	103	9	0.37	94	118	6	0.02
Arginine	992	109	84	<0.01	127	110	14	0.40
Proline	93	101	7	0.43	66	98	38	0.56

^1^Data are presented as means and average SEM, together with *P* value for the treatment effect.

Concentrations of amino acids in fetal and neonatal lamb plasma. At P140, fetuses carried by Arg ewes had greater plasma concentrations of ornithine whereas concentrations of taurine, threonine, glycine, methionine, tyrosine and histidine were decreased compared to fetuses from control ewes (Table 5). Within 2 h after birth, lambs from Arg ewes had greater concentrations of isoleucine and leucine compared to control fetuses, and tended to have greater plasma concentrations of valine, phenylalanine, ornithine, lysine and proline compared to controls.

**Table 5:** **Plasma concentrations of amino acids (μmol/L) 1 h after supplementation at d 140 of pregnancy (P140) of fetuses or 2 h post-birth of lambs from twin-bearing ewes either supplemented with arginine or saline (control) from P100 to P140 or parturition** ^**1**^

	Fetal and lamb plasma							
	P140				2 h post-birth			
	Arginine	Control	SEM	***P***value	Arginine	Control	SEM	***P***value
*n*	6	6			10	10		
Taurine	47	84	13	0.03	85	133	30	0.26
Aspartic acid	29	35	3	0.11	19	21	4	0.67
Threonine	616	763	47	0.04	624	608	82	0.83
Serine	530	567	37	0.49	310	257	51	0.40
Asparagine	43	37	9	0.64	112	74	25	0.23
Glutamic acid	122	88	22	0.28	65	55	8	0.36
Glutamine	405	372	22	0.30	431	362	44	0.28
Glycine	748	944	54	0.02	629	721	81	0.53
Alanine	414	399	26	0.63	500	437	66	0.47
Citrulline	218	200	37	0.51	124	71	25	0.15
Valine	350	342	62	0.82	413	262	59	0.09
Methionine	57	104	7	<0.01	84	77	15	0.57
Isoleucine	88	82	8	0.47	181	80	29	0.02
Leucine	172	173	14	0.90	404	175	60	0.02
Tyrosine	115	162	15	0.04	140	128	20	0.47
Phenylalanine	110	121	6	0.23	125	80	19	0.09
β-Alanine	37	50	12	0.45	88	61	21	0.37
Tryptophan	80	79	20	0.97	47	31	7	0.14
Ornithine	367	213	50	0.03	150	107	16	0.07
Lysine	152	192	24	0.24	222	108	41	0.07
Histidine	153	366	40	<0.01	303	333	50	0.77
Arginine	237	192	22	0.17	84	48	23	0.25
Proline	197	203	27	0.87	361	245	49	0.10

^1^Data are presented as means and average SEM, together with *P* value for the treatment effect.

### Concentrations of hormones and metabolites

At P140 Arg ewes had greater circulating concentrations of insulin and glucose but decreased concentrations of NEFA compared to control ewes. In contrast, no differences in circulating concentrations of hormones or metabolites were observed within 2 h post-birth between treatment groups (Table 6).

**Table 6:** **Plasma concentrations of hormones and metabolites 1 h after supplementation at d 140 of pregnancy (P140) or 2 h post-birth of twin-bearing ewes either supplemented with arginine or saline (control) from P100 to P140 or parturition** ^**1**^

	Ewe plasma							
	P140				2 h post-birth			
	Arginine	Control	SEM	***P***value	Arginine	Control	SEM	***P***value
*n*	6	6			5	5		
Insulin, ng/mL	0.33	0.05	0.07	0.02	0.68	0.56	0.18	0.74
IGF-1, ng/mL	130.6	138.5	12.3	0.66	136.4	197.2	32.0	0.22
Glucose, mmol/L	3.74	2.92	0.18	0.01	6.19	6.62	0.43	0.50
NEFA, mmol/L	0.33	0.76	0.14	0.05	0.35	0.40	0.10	0.20
Glycerol, mmol/L	0.05	0.07	0.01	0.11	0.08	0.07	0.02	0.82
Triglycerides, mmol/L	0.41	0.35	0.04	0.40	0.35	0.33	0.04	0.77

^1^Data are presented as means and average SEM, together with *P* value for the treatment effect.

At P140, fetuses from Arg ewes tended (*P* = 0.12) to have greater concentrations of insulin relative to controls. No differences in concentrations of IGF-1, glucose, NEFA, glycerol and triglycerides were found between fetuses from Arg ewes compared to controls (Table 7). Two h following birth, lambs from Arg ewes had elevated levels of insulin while no effect of Arg on the profiles of any other hormones and metabolites measured was observed (Table 7).

**Table 7:** **Plasma concentrations of hormones and metabolites 1 h after supplementation at d 140 of pregnancy (P140) of fetuses or 2 h post-birth of lambs from twin-bearing ewes either supplemented with arginine or saline (control) from P100 to P140 or parturition** ^**1**^

	Fetal and lamb plasma							
	P140				2 h post-birth			
	Arginine	Control	SEM	***P***value	Arginine	Control	SEM	***P***value
*n*	6	6			10	10		
Insulin ng/mL	0.12	0.06	0.03	0.12	0.39	0.10	0.11	0.03
IGF-1 ng/mL	94.4	93.1	7.0	0.91	100.1	95.1	10.4	0.62
Glucose mmol/L	1.51	1.52	0.39	0.98	4.49	3.47	0.51	0.15
NEFA mmol/L	0.10	0.14	0.06	0.29	1.50	1.47	0.11	0.91
Glycerol mmol/L	0.12	0.17	0.06	0.42	0.80	1.00	0.10	0.15
Triglycerides mmol/L	0.14	0.07	0.04	0.49	1.40	1.38	0.17	0.99

^1^Data are presented as means and average SEM, together with P value for the treatment effect.

### Lamb behavior at birth

A range of lamb behaviors were recorded at birth to provide estimates of lamb viability which, under farming conditions (i.e. when not continuously monitored and provided assistance when required) may influence lamb survival. Lambs born to Arg ewes tended to take longer to get to their knees (2.3 vs. 1.8 min; *P* = 0.07). A numerical difference was found for the percentage of lambs that were able to feed unassisted within 2 h of birth, such 80% of lambs born to Arg ewes were able to feed on their own vs. 57% of lambs born to control ewes (*P* = 0.12). A treatment by sex interaction (*P* = 0.02) was observed for the time taken to stand, such that male lambs born to Arg ewes took longer to stand than their control male counterparts (3.3 vs. 2.6 min; *P* = 0.01). No such treatment effect was observed between females (2.7 vs. 2.9 min; *P* = 0.49).

## Discussion

Intrauterine growth restriction as a result of multiple-pregnancy leads to decreased birth weight and increased mortality in offspring (Blickstein 2005; Gootwine et al. 2007). Previous studies have reported the effect of parenteral Arg supplementation from 100 to 121 days of pregnancy on fetal growth in multiple-bearing sheep (Lassala et al. 2010, 2011). However, to our knowledge, this is the first study to evaluate the effect of parenteral Arg supplementation of well-fed twin-bearing ewes from 100 d pregnancy to term, and the impact on circulating concentrations of amino acids and hormones in both the dam and fetus, body composition of the fetus in late pregnancy and lamb body weight and behavior at birth. The two key findings from this study are that maternal Arg supplementation increased brown fat stores of the fetuses at P140 and that Arg increased the birth weight of female, but not male, lambs without affecting maternal weight. Brown fat stores are important for thermoregulatory capacity of the neonate (Asakura 2004), which in combination with increased birth weight, may have implications for the survival of the newborn with importance for both agriculture and medicine.

Arginine is important for the synthesis of nitric oxide (NO) and polyamines which play a key role in placental angiogenesis and growth in mammals (Wu and Morris 1998; Wu et al. 2007; Sheppard et al. 2001; Ishikawa et al. 2007). Increased intrauterine growth of twin-born female lambs born to Arg ewes in this study contrasts the study of Lassala et al. (2011) where no effect of Arg on intra-uterine growth of twins was reported compared to their twin controls. Lassala et al. (2011) hypothesized that the lack of effect of Arg on twin and triplet intrauterine growth and development was dependent on factors such as the severity of uterine crowding, intra-uterine growth restriction and ketosis rather than Arg availability. However, two key differences are evident between the present study and the one by Lassala et al. (2011). Firstly, the present study had greater statistical power to detect a treatment effect because 11–12 ewes per treatment group were used compared to 3 control and 7 Arg twin-bearing ewes in the study by Lassala et al. (2011). Secondly, Arg was supplemented at the same dose-rate from 100 days pregnancy to term in our study, whereas Lassala et al. (2011) administered Arg from 100 to 121 d of pregnancy. We postulated that circulating levels of Arg in the maternal circulation becomes limiting to fetal growth in the last 3–4 weeks of pregnancy, the timeframe in which fetal growth restriction becomes evident (relative to singleton pregnancies) in twin-lambs (McCoard et al. 2000). At P140 there was no difference in fetal weight in response to Arg supplementation indicating that fetal growth was increased only in the last week of pregnancy. These observations highlight the potential for Arg supplementation of twin-bearing ewes to at least partially counter maternal constraint during late pregnancy.

The sex-specific effect of Arg on lamb birth weight is intriguing. In general, female lambs are 10-15% lighter at birth than male lambs (Afolayan et al. 2007), an effect that was ameliorated by Arg in this study. The potential for nutritional interventions to have sex-specific effects has been previously reported (Jaquiery et al. 2012). The ability to increase the birth weight of lambs has important implications for lamb survival and productivity (Afolayan et al. 2007; Everett-Hincks and Dodds 2008; McCoard et al. 2010; Greenwood et al. 2010). Further studies are required to validate these findings, and to evaluate the mechanism(s) mediating the potential sex-specific effect of Arg on intra-uterine growth of lambs.

The observed Arg-induced increase in peri-renal fat stores, the primary site of brown adipose tissue accumulation in the fetus, has significant potential impact to increase neonatal thermogenesis and survival. This is in agreement with the greater rectal temperatures observed in lambs born to Arg-ewes. The ability of the neonate to maintain core body temperature during cold stress is a key determinant of survival in sheep and other mammals (Symonds and Lomax 1992). In sheep and humans, this process is mediated in part by non-shivering thermogenesis whereby a large amount of heat is generated from metabolism of brown adipose tissue (Asakura 2004). Despite only constituting about 2% of the body weight of a newborn lamb, metabolism of brown adipose tissue accounts for about 50% of the heat generated by a newborn lamb (Symonds and Lomax 1992). Interestingly, the ability to increase intrauterine brown adipose tissue deposition in the ovine fetus through nutritional manipulation has been limited (Budge et al. 2000; Dietz et al. 2003; Encinias et al. 2004). Consistent with the observations in this study, Arg supplementation to either under-fed ewes (Satterfield et al. 2013) or diet-induced obese sheep (Satterfield et al. 2012) has previously been reported to increase fetal brown fat stores at 125 d of pregnancy indicating that Arg stimulates brown adipose tissue development. The potential for elevated Arg to influence the expression of key genes and signaling pathways involved in protein, lipid and energy metabolism in tissues such as adipose tissue (Jobgen et al. 2009a; Jobgen et al. 2009b; Jobgen et al. 2006) warrants further investigation.

Alterations in the Arg-NO and polyamine pathways may be important for the regulations of uteroplacental blood flow and intra-uterine growth restriction (Sooranna et al. 1995; Kwon et al. 2003; Wu et al. 2006). In particular, Arg is a common precursor for polyamines (regulator of DNA and protein synthesis) and nitrous oxide (signaling molecule and vasodilator) which play key roles in the regulation of placental angiogenesis and growth in many mammalian species (Wu and Morris 1998; Wu et al. 2007; Sheppard et al. 2001; Ishikawa et al. 2007). Therefore, Arg stimulation of the Arg-NO and/or polyamine pathways may represent a potential mechanism for the observed effects on brown adipose tissue and/or birth weight. Further, the ability for Arg to increase fetal growth leading to enhanced birth weight of female offspring, coupled with the ability for Arg to increase the birth weight of quadruplets (Lassala et al. 2011) and singletons from underfed ewes (Lassala et al. 2010), reinforces the notion that metabolic regulation may be effective in the amelioration or prevention of intra-uterine growth restriction (Wu et al. 2009; Wu et al. 2013; Wu et al. 2006).

The biological mechanism that mediates the effect of Arg on fetal growth is not clear. Increased brown fat stores and fetal growth of twins was associated with increased maternal circulating concentrations of Arg and ornithine at P121 and P140 with ornithine remaining elevated 2 h post-birth despite Arg administration being ceased up to 12 h prior to parturition. Based on current knowledge of the regulation of nitrous oxide synthesis in endothelial cells (Wu and Meininger 2002), it was postulated by Lassala et al. (2011) that Arg increases placental angiogenesis and uteroplacental blood flow thereby enhancing the transfer of oxygen and nutrients to the developing fetus. However, elevation of placental blood flow and angiogenesis would be expected to increase the transfer of almost all nutrients to the fetus as observed following administration of Sildenafil citrate (Satterfield et al. 2010). It is important to note that the blood samples were taken 1 hr post-infusion of the dam. Therefore it is possible that the samples were collected too early to detect changes in concentration of amino acids in the fetal plasma, or collected too late and Arg had been metabolized by the placenta and/or fetus. Ornithine is one of the end products of Arg breakdown by the enzyme arginase, creating urea. Thus, elevated concentrations of ornithine may reflect the breakdown of Arg administered in the previous bolus thereby facilitating ammonia detoxification via the urea cycle (Wu and Morris 1998). Two h post-birth, lambs from Arg ewes had greater concentrations of insulin, isoleucine and leucine whereas IGF-I and metabolite profiles remained unchanged. Arginine and leucine are amino acids that can promote insulin secretion (Brown et al. 2011), which in turn can positively affect fetal growth (Fowden 1992). Proline also tended to be increased in lambs born to Arg ewes. Proline has an important role in the synthesis of polyamines and pyroline-5-carboxylate which regulates cellular redox state thereby influencing conceptus development and growth (Wu et al. 2008). The changes in some, but not all amino acids in the maternal and fetal plasma, does not support the notion that Arg increases placental blood flow thereby enhancing overall nutrient supply to the fetus. However, blood flow was not measured directly in this study. The potential for Arg to influence placental development and/or function will be the subject of future investigations. In addition, whether elevated levels of insulin and glucose in maternal plasma influenced placental function, remains to be elucidated.

The increase in body condition score without a corresponding change in live weight in cohort 1 is intriguing and may indicate that Arg may enable ewes to more efficiently utilize nutrient intake to support fetal growth as well as increase body reserves. Increasing body condition during late pregnancy has the potential to support the dramatic increase in nutritional requirements of the growing fetuses in late pregnancy and to provide additional body reserves to support lactation post-birth. In support of improved efficiency of utilization of dietary nutrients in Arg ewes, maternal NEFA concentrations in plasma decreased about 50% relative to controls. This suggests reduced mobilization of body reserves, while feed intake was unaffected and body condition score either remained unchanged (cohort 2) or was increased (cohort 1). These observations contrast previous studies where Arg supplementation of multiple-bearing ewes had no effect on NEFA profiles (Lassala et al. 2011) or increased NEFA concentrations in under-fed ewes (Lassala et al. 2010). Further research would be required to determine the relevance of these findings.

To survive, newborn lambs need to quickly get access to colostrum to maintain body temperature. Colostrum intake is influenced by lamb vigor, maternal colostrum production and bonding between the mother and offspring (Nowak and Poindron 2006). Whereas lambs from Arg ewes took longer to get to their knees or stand, the increased proportion of lambs that fed unassisted within 2 h of birth may indicate the potential for increased colostrum intake and thus survival compared with lambs from control ewes. Ewes that mobilize less body fat during pregnancy give birth to lambs that stand and suckle faster (Dwyer et al. 2003). The reduction in plasma NEFA in Arg ewes relative to controls at P140 is consistent with reduced mobilization of body reserves, coupled with the potential benefit of Arg on behaviors that are important for improving lamb survival is consistent with this notion. Lamb survival and maternal behavior traits have limited heritability (Everett-Hincks et al. 2005) indicating environmental and management strategies are required to improve lamb survival. Supplementation with Arg in mid-late pregnancy may therefore be a potential strategy to be investigated further in future studies.

In conclusion, Arg supplementation of twin-bearing ewes from P100 to birth improved brown fat stores in the fetuses at P140, birth weight of female lambs, and neonatal behaviors that may enhance survival. The potential for Arg to enhance the survival of multiple-born lambs has important implications for sheep production. Furthermore, using sheep as a model for humans, the findings in this study may contribute to the development of therapeutic interventions for women carrying twins.

## Abbreviations

Arg: L-Arginine
d: Day
h: Hour.
